# Development of the Post Cardiac Surgery (POCAS) prognostic score

**DOI:** 10.1186/cc13017

**Published:** 2013-09-24

**Authors:** Eduardo Tamayo, Inma Fierro, Juan Bustamante-Munguira, María Heredia-Rodríguez, Pablo Jorge-Monjas, Laura Maroto, Esther Gómez-Sánchez, Francisco Jesús Bermejo-Martín, Francisco Javier Álvarez, José Ignacio Gómez-Herreras

**Affiliations:** 1 Department of Anaesthesiology and Reanimation, Hospital Clinico Universitario de Valladolid, Avda Ramón y Cajal 3, 47005 Valladolid, Spain; 2 Department of Pharmacology and Therapeutics, Facultad de Medicine, University of Valladolid, Avda Ramón y Cajal 3, 47005 Valladolid, Spain; 3 Department of Cardiovascular Surgery, Hospital Universitario de La Princesa, Calle Diego de León nº 62, 28006 Madrid, Spain; 4 Department of Cardiac Surgery, Hospital Clinico Universitario de Valladolid, Avda Ramón y Cajal 3, 47005 Valladolid, Spain; 5 Infection & Immunity Unit, Hospital Clínico Universitario de Valladolid, SACYL/IECSCYL, Avda Ramón y Cajal 3, 47005 Valladolid, Spain

## Abstract

**Introduction:**

The risk of mortality in cardiac surgery is generally evaluated using preoperative risk-scale models. However, intraoperative factors may change the risk factors of patients, and the organism functionality parameters determined upon ICU admittance could therefore be more relevant in deciding operative mortality. The goals of this study were to find associations between the general parameters of organism functionality upon ICU admission and the operative mortality following cardiac operations, to develop a Post Cardiac Surgery (POCAS) Scale to define operative risk categories and to validate an operative mortality risk score.

**Methods:**

We conducted a prospective study, including 920 patients who had undergone cardiac surgery with cardiopulmonary bypass. Several parameters recorded on their ICU admission were explored, looking for a univariate and multivariate association with in-hospital mortality (90 days). In-hospital mortality was 9%. Four independent factors were included in the POCAS mortality risk model: mean arterial pressure, bicarbonate, lactate and the International Normalized Ratio (INR). The POCAS scale was compared with four other risk scores in the validation series.

**Results:**

In-hospital mortality (90 days) was 9%. Four independent factors were included in the POCAS mortality risk model: mean arterial pressure, bicarbonate ratio, lactate ratio and the INR. The POCAS scale was compared with four other risk scores in the validation series. Discriminatory power (accuracy) was defined with a receiver-operating characteristics (ROC) analysis. The best accuracy in predicting in-hospital mortality (90 days) was achieved by POCAS. The areas under the ROC curves of the different systems analyzed were 0.890 (POCAS), followed by 0.847 (Simplified Acute Physiology Score (SAP II)), 0.825 (Sepsis-related Organ Failure Assessment (SOFA)), 0.768 (Acute Physiology and Chronic Health Evaluation (APACHE II)), 0.754 (logistic EuroSCORE), 0.714 (standard EuroSCORE) and 0.699 (Age, Creatinine, Ejection Fraction (ACEF) score).

**Conclusions:**

Our new system to predict the operative mortality risk of patients undergoing cardiac surgery is better than others used for this purpose (SAP II, SOFA, APACHE II, logistic EuroSCORE, standard EuroSCORE, and ACEF score). Moreover, it is an easy-to-use tool since it only requires four risk factors for its calculation.

## Introduction

Mortality in heart surgery with cardiopulmonary bypass (CPB) is high, and both patients and relatives want to know the risk of mortality that such operations entail. In order to be able to forecast the outcome after cardiac surgery, various authors [1-6] have tried to establish a risk stratification for operative mortality after cardiac operations in adult patients and have developed risk scales.

In general, while the different risk scales used in cardiac surgery have been developed by including differing types and numbers of variables (ranging from 3 to 17), they all share, as a common strategy, that of only considering preoperative or surgery procedural variables as risk factors [7]. These risk scales/scores are intended to be used for all adult cardiac surgical procedures, considering them to be operative (within 30 days) mortality. In Europe, one of the most used scales is the Additive EuroSCORE [4], although it has been felt that this model has some drawbacks. First, an over-prediction of mortality may occur in low-risk cases [4,8,9]. Conversely, the additive model has also been shown to be a poor predictor of mortality in higher-risk patients [5,9]. To help improve the accuracy of the EuroSCORE, a logistic model, Logistic EuroSCORE [5] has become available. It was hoped that this would increase accuracy when predicting mortality in higher-risk patients [5]. However, there still is some concern that the logistic model over-predicts mortality in many risk groups [4,10]. More recently, a simpler scale has been developed, the ACEF score [6], which only takes three variables into consideration (age, creatinine and ejection fraction).

Although these scales are used in normal clinical practice, it has been pointed out that their main limitation is that they use parameters measured before surgical intervention and that they do not take into account the response of the organism to surgical aggression and CPB and the possible complications that could arise during surgery (for example, bleeding, acute myocardial infarction, low cardiac output). It is for these reasons that scales based on variables measured upon admittance to the ICU could be considered to be better indicators for predicting mortality. However, general severity systems, such as the Acute Physiology and Chronic Health Evaluation (APACHE II) [11], Simplified Acute Physiology Score II (SAPS II) [12], and Mortality Probability Models (MPM II) [13], when applied to heart surgery patients, do not work well in predicting in-hospital mortality and their precision is lower than that of those based on preoperative scores [14]. Studies focused on hemodynamic parameters and organism functionality upon admittance to ICU after heart surgery have identified several mortality-predictive variables for heart surgery patients, such as hyperlactatemia, bicarbonate, heart rate and creatinine [15-17].

In clinical practice, the scales that are available are not all that could be desired for predicting the risk of mortality after cardiac surgery [9]. Our study was based on the hypothesis that it was necessary to develop a specific risk score for cardiac surgery on the basis of organism functionality parameters upon admittance to ICU; we believed that these could predict the mortality of cardiac surgery patients better than the existing scales based on preoperative data [2-6,14] and than the scales that measure variables upon ICU admittance but are not specifically designed for cardiac surgery patients [11-13].

The present study was aimed at: 1) finding associations between the general parameters of organism functionality upon ICU admission and operative mortality following cardiac operations; 2) developing a Post Cardiac Surgery-POCAS-Scale; 3) defining operative risk categories; and 4) validating an operative mortality risk score.

## Material and methods

### Study design

A prospective open-cohort study was designed to assess risk factors for mortality after cardiac surgery with CPB, carried out between January 2009 and January 2011, in the Hospital Clínico Universitario, Valladolid (Spain), a level III healthcare medical center with 800 beds. Within this cohort study, patients were classified into two groups: survivors and non-survivors (in-hospital mortality being within 90 days).

The Hospital Clínico Universitario Valladolid Ethics Committee approved the study and waived the need for an informed consent from the patients. However, the patients had given their written consent to store their data in an anonymous form in the hospital database for scientific treatment at the time of their hospital admission, in accordance with the Spanish law regulating personal privacy matters.

### Study population

During the period of the study, all adult patients (18 years of age and older) scheduled for cardiac valve and/or coronary surgery with CPB were included. Transplant patients were excluded.

### Postoperative care

At the end of surgery, patients were transferred to the ICU, where they were treated according to a standard regimen. Hemodynamic values were assessed at a heart rate of 70 to 80 beats/minute and mean arterial pressure at 65 to 80 mmHg. Inotropic support depended on the individual status of the patient. Basic IV-fluid administration consisting of 0.9% NaCl and gelatin was infused. Fluid balance, rectal temperature and peripheral temperature (measured on the back of the foot) were recorded every hour. Lungs were ventilated with 60% oxygen using volume-controlled ventilation and a tidal volume of 10 ml/kg with 5 cm H_2_O of positive end-expiratory pressure. Arterial blood gas was analyzed by standard techniques using an automated analyzer and anesthesia induction at 4-hour intervals for 24 hours after termination of CPB. All patients were extubated in the ICU when the Tobin index (respiratory rate (spontaneous)/tidal volume (L) was <105 [18], PaO_2 _was >60 mm Hg, FIO_2 _was <0.4, continuous positive airway pressure was <5 mbr, PCO_2 _was 50 mm Hg, and arterial pH was >7.35.

### Study variables

#### Outcome variables

The primary outcome variable was 90-day in-hospital mortality.

#### **I**ndependent variables

Preoperative, intraoperative and postoperative potential risk factors, risk score, and parameters at admission to the ICU (see below) were defined as independent variables.

The preoperative risk for operative mortality was evaluated by means of the Additive and logistic EuroSCOREs [4,5] and the ACEF score [6]. The postoperative risk was evaluated using the SAP II [12], SOFA [19] and APACHE II scores [11].

#### Preoperative values

The preoperative values were age, sex, weight, height, hypertension, atrial fibrillation, diabetes mellitus, obesity, smoking, drinking alcohol, hepatic disease, respiratory disease, chronic renal failure, immunosuppression, previous cardiac surgery, and emergent surgery.

#### Perioperative values

The perioperative values were left ventricle ejection fraction, valve, coronary artery bypass graft (CABG), valve + CABG, total CPB time and aortic cross-clamp time.

#### Postoperative values

The postoperative values were duration of mechanical ventilation, use of vasopressor drugs, reintubation, multiple blood transfusions, acute renal failure, re-intervention, intra-aortic balloon pump, pneumonia, surgical site infections, preoperative hospitalization, mean ICU stay, duration of hospitalization after ICU and total duration of hospitalization.

### Parameters at admission to the ICU

The following data were collected immediately upon admission to the ICU after cardiac operations: pH, bicarbonate (HCO_3_-), partial pressure of carbon dioxide (PCO_2_), ratio of partial pressure of arterial oxygen to fraction of inspired oxygen (PaO_2_/FiO_2_)_, _core temperature, leukocyte, C-reactive protein, procalcitonin, lactates, central venous oxygen saturation (ScvO_2_), heart rate, mean arterial pressure, glucose, creatinine, hematocrit, Na, K, troponin T (TnT), creatine kinase-MB (CK-MB), International Normalized Ratio (INR), activated partial thromboplastin time ratio (aPTTr) and platelets. In all cases, hemodynamic parameters (heart rate and mean arterial pressure) were registered 30 minutes after admission to the ICU and once the patient had reached hemodynamic stability.

### Statistical analysis

Statistical analyses were performed using the Statistical Package for Social Sciences (SPSS) version 19 and Epidat version 3.1. Differences between the two groups were examined using Pearson's Χ^2 ^test for categorical data and Student's t-test for continuous variables. Mortality percentages are presented with 95% confidence interval (CI) using Wilson's method. A value of *P *<.05 was considered significant for all statistical tests.

To assess the associations between the general parameters of organism functionality at ICU admission and operative mortality within 90 days following cardiac operations, all organism functionality variables determined on admission to the ICU and which were significant in the univariate analysis were subsequently tested for accuracy with a receiver-operating characteristics (ROC) analysis, with the area under the curve (AUC) as a measurement of accuracy, according to the methodology used and validated in other studies [6].

The variables with areas under the ROC curve less than 0.5 were transformed into their inverses, in order to observe the same classification criteria with all the variables [20]. The variables with the best AUC values (AUC >0.7) were used in a subsequent multivariable logistic analysis. In order to adjust the coefficients with greater precision (to avoid losing cases with the rest of the variables that were not significant), the logistic regression was repeated with the variables that had been significant in the previous analysis. This logistic regression model was tested for calibration with a Hosmer-Lemeshow Χ^2 ^and the possible multicollinearity of the model was checked with an analysis of tolerance and inflation statistics.

The above model was subsequently translated into a mortality risk score, developed on the basis of the respective weights of the significant variables (based on their regression coefficients). Using the B coefficients of the logistic regression, the logit function (Z) was constructed and the Z values and each one of the products B_i_X_i _were saved as new variables in the database. Z = B_0 _+ B_1_X_1 _+ B_2_X_2 _+......+ B_n_X_n._

ROC curves were generated for each of these new variables and used to identify the cutoff points that defined the ranges of scores for 90-day in-hospital mortality for each predictor. The cutoff points of the ROC curves for scores (B_i_X_i_) were correlated with the corresponding values of the original variables. The corresponding B_i_Xi average value was allocated as a point score to each resultant range of values of the original variable Xi (predictor) within that particular grouping. The sum of weighted risk scores was then calculated for each patient. Initial operative risk categories for 90-day in-hospital mortality were calculated from the cutoff points defined by the ROC curve obtained with the overall point score.

Differences between predicted and observed mortality rates were explored for different risk classes, determined by their cutoff points on the ROC curve, by comparing predicted/observed event rates with 95% (CIs). In the validation of the new operative mortality risk score scale, the patient received a risk assessment using the newly developed score, plus four previously established mortality scores: the additive and logistic EuroSCOREs, ACEF, SAP II, SOFA and APACHE II. ROC analyses were applied to establish the accuracy and calibration of each risk score scale. From the ROC analysis, the best cutoff values for each score were identified at the point where the sum of sensitivity and specificity was the highest according to the Youden index ((sensitivity+specificity)-1). Sensitivity, specificity, and positive and negative predictive values for each cutoff value in each risk score were calculated.

Comparisons between ROC AUCs were carried out for the paired data. All empirical curves were constructed based on tests performed on the same individuals and the correlated nature of the data was taken into account for the statistical analysis on differences between ROC AUCs [21,22].

## Results

### Patient characteristics

#### Characteristics of the patients included in the study and their survival up to hospital discharge after cardiac surgery

During the period of the study, a total of 920 adult patients were operated on at the cardiac surgery center concerned. In the operating theater, 11 patients died during the operation. A total of 909 patients were admitted to the ICU and there were 80 in-hospital (within three months) deaths (8.80%, 95% CI: 6.96% to 10.65%).

#### Differences between surviving and non-surviving patients with cardiac surgery

Survivors were younger than non-survivors (Table 1). Non-survivors more frequently showed a medical history of previous respiratory diseases, chronic renal failure and emergent surgery. Non-survivors also showed a lower left ventricular ejection fraction, had undergone valve+CABG surgery more frequently, had spent longer on CPB and had spent more time with aortic cross-clamping. During the postoperative period, non-survivors needed re-intubation much more frequently and more often showed various types of postoperative complications: acute renal failure, re-intervention, intra-aortic balloon pump, pneumonia, preoperative hospitalization and mean ICU stay. As a result, the additive and logistic EuroSCOREs and the ACEF, SAP II, SOFA and APACHE score scales were significantly higher in non-survivors (Table 1).

### Predictors of in-hospital mortality within 90 days in patients with cardiac surgery

Surviving and non-surviving patients differed in all parameters measured on their admission to the ICU except in core temperature, glucose, potassium, and platelet counts (Table 2).

A ROC analysis was performed for each one of these variables identified in the univariate analyses, with estimation of the AUC and 95% CI. The variables with the best AUC were lactate, mean arterial pressure (MAP), bicarbonate, INR, creatinine, procalcitonin, leukocyte, heart rate and C-reactive protein. These nine variables were subsequently entered into a multivariate logistic model (forward stepwise). In this analysis, four variables (INR, MAP, lactate, and bicarbonate) were confirmed to be independent predictors of mortality. We performed the multivariate logistic model again including only these four variables but, with MAP and bicarbonate, a mathematical transformation was used (1000/MAP and 1000/bicarbonate). These transformations did not affect the model significantly and made it possible to find positive coefficients (B), in order to create an additive scale with only positive contributions.

Mortality risk (Table 3) was directly correlated with INR (odds ratio (OR) = 2.79, 95% CI: 1.29 to 6.06), 1000/MAP (OR = 1.36, 95% CI: 1.24 to 1.49), lactate (OR = 1.02, 95% CI: 1.01 to 1.04), and 1000/bicarbonate (OR = 1.04, 95% CI: 1.01 to 1.06). This multivariate model was well calibrated (Table 3; see Hosmer-Lemeshow value), and multicollinearity was excluded within these four variables (Table 3; see tolerance values).

### Development of an operative mortality risk score scale: the POCAS scale

**Figure 1:**
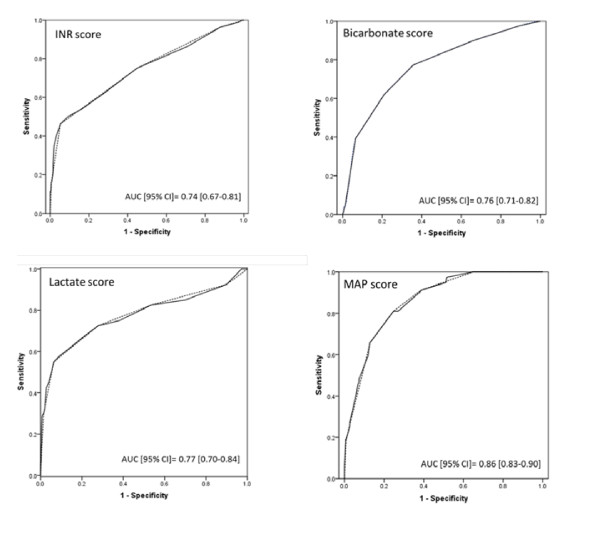
**ROC Curves for Individual Scores (B_i_X_i_) Used in 90-Day In-Hospital Mortality Prediction in Solid Line (established cutoff points in dotted lines)**.

The above model, with these four variables, was subsequently translated into a mortality risk score: the POCAS score (Table 4). The mortality risk score was developed on the basis of the respective weights of the significant variables (based on their regression coefficients; Table 3) and cutoff points were established by analyzing the ROC curve coordinates for mortality risk according to the products BiXi. The cutoff points of the ROC curves define the different points scale for each of the BiXi variables (Figure 1). We assigned the median value of the corresponding BiXi value in that same range (for example, 'Bicarbonate score' = 22 in the above 'Bicarbonate' range for BiXi = 0.033*('1000/Bicarbonate')) to the interval defined by the minimum-maximum of each original variable in that group (for example, 'Bicarbonate' 14.6 to 20.3 mEq/L).

**Figure 2:**
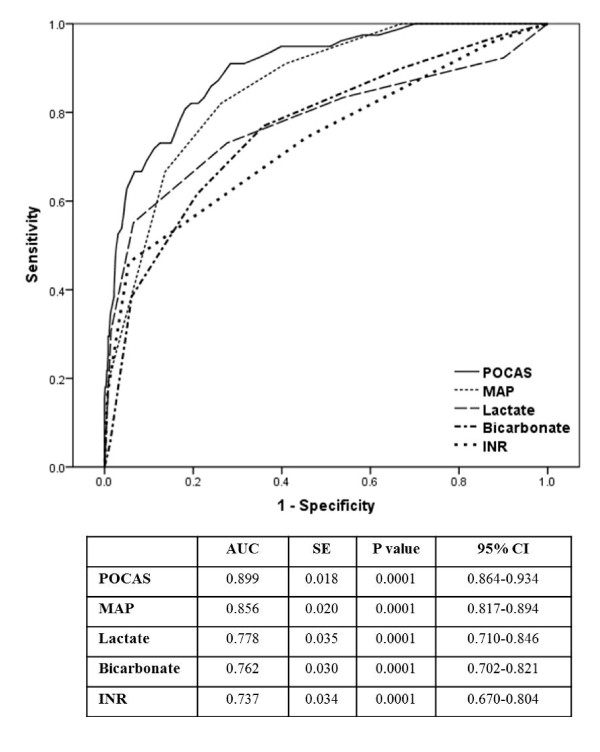
**POCAS ROC Curves, Lactate, Bicarbonate, MAP, and INR Score for Predicting 90-Day In-Hospital Mortality (AUC, Area under Curve)**.

The new scale was applied to the database and a total point score was calculated: POCAS (total score) = lactate score + INR score + MAP score + bicarbonate score. The POCAS score created with these four variables was tested for significance, accuracy, and calibration using a logistic regression model with Hosmer-Lemeshow _X_^2 ^and ROC analysis (Table 5). The POCAS was significantly (*P *<.001) correlated with operative mortality and demonstrated very good calibration (Hosmer-Lemeshow test, Table 5, _X_^2 ^= 3.60; *P *= .89). The accuracy was moderately good, with an AUC of 0.90, 95% CI: 0.86 to 0.93 (Figure 2).

### Operative risk categories

The probability of 90-day in-hospital mortality, including predicted and observed 90-day in-hospital mortality, was calculated for each of the groupings determined by the cutoff points of the ROC curve for the total score (Figure 2). On this basis, patients were classified into seven operative risk categories, which were later re-grouped into four: low (≤90 points), medium (91to105 points), high (106 to 128 points) and very high (>128 points). The observed 90-day in-hospital mortality was 2.3%, 15.6%, 55.8%, and 100%, respectively. The % probability (95% CI) for these categories was 2.3 (2.2 to 2.5), 16.3 (15.3 to 17.4), 56.2 (51.9 to 60.6), and 95.0 (92.8 to 97.2), respectively (Table 6).

### Validation of the model

The patients included in the study received a risk assessment using the new POCAS score scale and its performance was compared with four previously-established mortality risk scores: the additive and logistic EuroSCOREs, the ACEF score, SAP II, SOFA and APACHE II. Table 7 reports the sensitivity, specificity, and positive and negative predictive values for the cutoff values identified. The POCAS had a very good accuracy in all subsets of the population, with AUC = 0.9. SAP II was the second-best predictor for accuracy in the overall population (AUC = 0.84) in patients after cardiac surgery, followed by SOFA (AUC = 0.82) and APACHE II (AUC = 0.76). Significant differences were found between the ROC curves of the POCAS scale and each of the other scales (Table 7).

## Discussion

The most relevant findings of this research are as follows: 1) The results of this study confirmed the validity of the proposed hypothesis, according to which a mortality risk score developed on the basis of the hemodynamic parameters and organism functionality upon admittance to an ICU after cardiac surgery would allow for the establishment of a risk model with greater accuracy than that of the scales that had used preoperative risk factors, such as the additive and logistic EuroSCOREs [4,5], ACEF [6], or APACHE II [11]; 2) The scale that we designed (POCAS) showed the greatest area-below-curve of all the scales evaluated in this study (additive and logistic EuroSCOREs, ACEF, SOFA, SAP II, APACHE II); 3) In theory, it seems quite logical to think that the organism functionality variables upon ICU admittance after a cardiac surgery operation should be strongly correlated with patient mortality risk. This study has demonstrated the possibility of correlating several of these variables with 90-day mortality by means of a well-calibrated model, which, at the same time, is easy to use since it only uses four risk factors in its calculation; and 4) Given the enormous complexity of patients undergoing cardiac surgery and the great variety of possible complications that may arise, the use of organism functionality variables upon ICU admittance offers the possibility of using a widely-applicable, highly-accurate scale, regardless of the intra- and post-operative complications of each patient.

Our study presents a 90-day in-hospital mortality rate of 8.8%, which might seem high if compared to other studies performed outside Spain, such as, for example, Ranucci *et al*., (3.3%, operative deaths); Badreldin *et al*. (3.6% overall mortality) or Roques *et al*. (4.8%, overall hospital mortality). However, this observed mortality rate is comparable to other Spanish studies. Curiel-Balsera *et al*. performed a study with data obtained from The Registry of Adult Cardiac Surgery Platform ARIAMAndalusi, and which included data from 11 hospitals in the autonomous community of Andalusia between 2008 and 2011, in a prospective manner [23]. A total of 4,548 patients with cardiac surgery were included in the study, intra-ICU mortality was 7.7% and 30-day mortality was 9.3%. Rodríguez-Rieiro *et al*. in a study designed to determine whether there is a difference between expected and observed mortality rates after coronary artery bypass grafting (CABG) in Spanish autonomous regions, included all patients registered in a minimum basic data set (MSBD) undergoing CABG between 2000 and 2004. The expected Spanish in-hospital mortality rate after CABG was 7.68 and the observed rate was 7.69 deaths per 100 operations [24,25]. Scales based on ICU admittance variables may well be better predictors of mortality; however, APACHE II [11], SAPS II [12], and MPM II [13] do not work very well in predicting in-hospital mortality when applied to heart surgery patients and their accuracy is lower than that of the preoperative Parsonnet model [14]. This is probably because they include parameters that are strongly affected by the very nature of cardiac surgery with CPB. In this environment, leukocytosis is principally due to the inflammatory response effects of CPB, central temperature may be affected by the level of hyperthermia during CPB, and the Glasgow Coma Scale cannot be evaluated upon ICU patient admittance. Apart from this, intraoperative factors may modify the preoperative risk stratification: changes in operative planning, poor operative results, inadequate myocardial protection and so on. All these and other serious intraoperative factors could increase the risk of operative mortality to values above those indicated by preoperative risk models. On the other hand, a heart surgery operation without complications and with correct handling could reduce the risk of operative mortality to levels lower than expected. For these reasons, the POCAS scale in this study proved to be more accurate than those of the additive and logistic EuroSCOREs, ACEF, SAP II and APACHE II [11-13].

The CASUS scale [26] is the only one designed for predicting cardiac surgery mortality by the use of ten variables measured upon ICU admission. In several studies, it has been concluded that this is a good scale for predicting cardiac surgery mortality and its usage has been recommended [8]. This scoring system has not yet been externally validated in multicenter studies and, accordingly, has not yet gained much popularity. Furthermore, the use of the SOFA score [19] has been validated in cardiac surgery as a good mortality predictor, as much upon ICU admittance as on an evolutionary basis [27]. CASUS and SOFA are reliable ICU mortality risk stratification models for cardiac surgery patients. SAPS II and APACHE II did not perform well in terms of calibration and discrimination statistics [28]. It would, therefore, possibly be of great interest to compare the heart surgery mortality prediction score of the POCAS score with those of the CASUS [26] and SOFA scores [19]. Our risk model, POCAS, when compared with the SOFA score, demonstrated greater accuracy. However, we could not compare the CASUS score because this scale assesses neurologic state in four degrees (confused, conversation, sedated diffuse, neuropathy) and in our database we have used the Glasgow scale.

The variables used for the POCAS scale (lactate, MAP, bicarbonate and INR) have also been identified by other authors [11-13,15-17,29] as predictors of mortality. MAP is used by all the principal scales that evaluate ICU admittance parameters except for MPM II; bicarbonate is included in APACHE II and SAP II; INR is only included in MPM II. The POCAS scale is the only one that includes blood lactate levels, although the association of elevated lactate values at ICU admission with hospital/operative mortality has already been highlighted in other studies [17,30,31].

Shock is best defined as inadequate tissue perfusion and, thereby, an impairment of oxygen delivery. Regional hypoxia results in anaerobic metabolism that produces two ATP molecules (versus 36 in aerobic metabolism) and pyruvate, which is converted into lactic acid. Prolonged, severe tissue hypoperfusion results in the generation of large quantities of hydrogen ions (H^+^) from lactic acid. Plasma bicarbonate acts as a buffer for serum hydrogen ions released during anaerobic metabolism and, therefore, decreases as the acidosis worsens. Traditional hemodynamic markers, such as blood pressure, have been used to guide resuscitation in case of shock. Lactate and bicarbonate have been demonstrated to be metabolic indicators of the severity of shock and related to mortality [15,32,33]. MAP is a key clinical factor in the scoring system and its values can be altered during patient transfer from the operating room to the ICU. For this reason, and so that the MAP values were the most representative of the patient's hemodynamic situation upon ICU admission, the MAP values from 30 minutes after ICU admission were included in the POCAS scale.

The INR is a liver functionality variable that is included in more specific scales, such as that of Child-Pugh [34]. In a prospective, multicenter, observational, cohort study that they participated in, it was observed that, of the 1,923 patients admitted to the ICU, 30% developed abnormal INR values (defined as an INR >1.5). Most INR abnormalities were minor and short-lived (73% of the worst INR values 1.6 to 2.5). In all regression models, there was a strong independent association between abnormal INR values and greater ICU mortality (*P *<.0001), particularly when INR increased after ICU admission [29].

The use of only four variables for the risk assessment of such a group of patients, in which complexity is high, could give the impression that other variables that could also be important have been overlooked. Moreover, it could seem that by taking into account a greater number of factors in the risk model a more accurate risk estimation would necessarily be obtained. In this respect, there are a number of considerations that should be remembered in order to understand the methodological development better.

The problem of overfitting: When numerous variables are included in an attempt to 'control' or 'adjust' data, the accuracy of the results can be threatened [35]. From a statistical point of view, it is ideal to be parsimonious in the choice of independent variables. To avoid including too many independent variables, some authors even suggest 'clustering', grouping together variables with a similar clinical significance [36]. Wells and colleagues [37] concluded that 'less is more in multivariable analysis.' Of course, if a simple model can explain a phenomenon with the same level of accuracy as that of a more complex model, it thus agrees with the 'law of parsimony' or the concept of 'Occam's razor.' It is much more efficient and more applicable. Following the methodology of other studies [6], in the POCAS scale, we performed a ROC analysis on each of the variables that were significant in the univariate analysis to select the initial variables.

The problem of risk factor definition: In the choice of risk factors, it is preferable to include continuous variable scales rather than categorical binary ones because, in this way, the values will not be submitted to personal subjectivity. The four risk factors used in the POCAS are continuous variables. The POCAS scale does not include categorical binary risk factors, such as chronic obstructive pulmonary disease, cerebral-vascular illness, extra-cardiac arteriopathy, or unstable angina, included in other risk scores (EuroSCORE, APACHE II) [4,5,11].

Categorical binary risk factors need to be defined to be included in a model. Such definitions are clearly established in each risk score, but they are subject to a certain degree of personal interpretation. Therefore, it is possible for different observers to offer different interpretations giving, as a result, different final risk score point ratings, as has been shown previously by other authors [38].

The problem of multicollinearity: Multicollinearity is defined as the intercorrelation between independent variables included in a risk model. In the POCAS score, collinearity could be thought to exist between the MAP, lactate and bicarbonate variables, due to their being variables that measure tissue perfusion. However, they have been tested for multicollinearity and no intercorrelation was found. It was not possible to analyze the intercorrelation of other risk scores, but including a large number of independent variables (EuroSCORE: 17 variables and APACHE II: 17 variables) [4,5,11] increases the risk of multicollinearity.

Inclusion/exclusion of risk factors: It is understood that a simple risk score based on lactate, MAP, bicarbonate and INR excludes many other possible risk factors. The exclusion of all the co-morbidities and characteristics of the operation could be a reason for concern. Nevertheless, if the co-morbidities included in other risk scores [4-6,13] and excluded by POCAS are taken into account, the truth is that there is no general agreement upon which should be included and which, excluded. Of the risk scales based on organism functionality variables developed over ICU admittance variables (APACHE II, SAPSII, MPM II, SOFA and CASUS) [11-13,19,26], three also include preoperative variables and only SOFA and CASUS [19,26] only use organism functionality risk factors.

### Study limitations

Perhaps the greatest limitation of this present study is that it was based on data obtained from one single medical center and, therefore, it is probable that patient selection and the carrying out of procedures (such as perioperative handling) could vary between different cardiac surgery units and be important determinants in mortality. The POCAS scale should therefore be repeated through a multicenter study.

### Application of the results

The POCAS scale is easy to use and permits a re-evaluation of the risk of mortality after a short period of time (one to two hours) after the operation, supplying more accurate information. Finding a point score on the POCAS scale in the operative risk categories of high or very high suggests a 90-day mortality of 55.8% and 100%, respectively; this information is important for the patient's relatives in that they may be made aware of it when they are informed of the patient's clinical condition. It is also important for doctors, given that such data will indicate to them that the patient will need extra-special care and closer attention during the postoperative period (Swan-Ganz catheter and/or transesophageal echocardiography) both to identify the nature and to quantify the severity of possible cardiac insufficiency.

## Conclusions

This study has shown that this new system to predict the operative mortality risk (POCAS) of patients undergoing cardiac surgery, based on organism functionality variables, is better than others used for this purpose (SOFA, SAP II, APACHE II, logistic EuroSCORE, standard EUROSCORE, and ACEF score). Moreover, it is an easy-to-use tool because it only requires four easily-measured risk factors, determined upon ICU admittance, to calculate it and it makes it possible to establish a 90-day, in-hospital mortality risk.

## Key messages

• The organism functionality parameters on admission to ICU reflect changes which have occurred during the surgical procedure and allow development of more precise postoperative surgical risk models than scales using only preoperative parameters.

• POCAS is a simple postoperative scale, easy to apply and reproduce, which requires only four factors for its calculation. It could be an important complement to traditional risk scales in preoperative risk estimation.

## Abbreviations

ACEF: Age Creatinine Ejection Fraction score; APACHE: Acute Physiology and Chronic Health Evaluation; AUC: area under curve; CABG: coronary artery bypass graft; CI: confidence interval; CPB: cardiopulmonary bypass; ICU: intensive care unit; INR international normalized ratio; MAP: mean arterial pressure; MPM: mortality probability models; POCAS: post cardiac surgery; ROC: receiver-operating characteristics; SAPS: Simplified Acute Physiology Score; SOFA: Sepsis-related Organ Failure Assessment.

## Competing interests

The authors declare that they have no competing interests.

## Authors' contributions

ET, IF and JBM conceived the idea, designed the protocol and supervised the analysis of the results. ET, FJA and JIGH contributed to obtaining the funding, oversaw the study, and acted as guarantors for the report. MHR, LM, PJM, EGS and FJBM helped in the conception of the study and the design of the protocol, supervised the recruitment of patients, collected the clinical material and were responsible for statistical analysis. ET, IF, JBM, FJA, EGH, PJM and LM developed and supervised all work and analyzed the results. ET wrote the report in collaboration with FJA, JBM, IF, JIGH, JFBM and the other authors. All authors read and approved the final manuscript.
