# Erratum to: Development of the Post Cardiac Surgery (POCAS) prognostic score

**DOI:** 10.1186/s13054-015-1117-0

**Published:** 2015-11-09

**Authors:** Eduardo Tamayo, Inma Fierro, Juan Bustamante-Munguira, María Heredia-Rodríguez, Pablo Jorge-Monjas, Laura Maroto, Esther Gómez-Sánchez, Francisco Jesús Bermejo-Martín, Francisco Javier Álvarez, José Ignacio Gómez-Herreras

**Affiliations:** Department of Anaesthesiology and Reanimation, Hospital Clinico Universitario de Valladolid, Avda Ramón y Cajal 3, 47005 Valladolid, Spain; Department of Pharmacology and Therapeutics, Facultad de Medicine, University of Valladolid, Avda Ramón y Cajal 3, 47005 Valladolid, Spain; Department of Cardiovascular Surgery, Hospital Universitario de La Princesa, Calle Diego de León nº 62, 28006 Madrid, Spain; Department of Cardiac Surgery, Hospital Clinico Universitario de Valladolid, Avda Ramón y Cajal 3, 47005 Valladolid, Spain; Infection & Immunity Unit, Hospital Clínico Universitario de Valladolid, SACYL/IECSCYL, Avda Ramón y Cajal 3, 47005 Valladolid, Spain

After Publication of the original article [1] it has been brought to our attention that none of the tables referred to in the text have been included in the article. Tables 1, 2, 3, 4, 5, 6 and 7 are now displayed below.

**Table 1:** Patient population profile (Survivors and non-survivors)

Variable	Survivors (*N* = 829)	Non survivors (*N* = 80)	*P* value
Preoperative factors			
Age, years	66.3 ± 11.2	68.9 ± 11.3	**0.05**
Sex, M/F male gender	574 (69.2)	48 (60)	0.09
Peso, kg	72.7 ± 15	71.9 ± 11.4	0.69
Talla, cm	163.3 ± 9	162.3 ± 8.9	0.34
Hypertension	449 (54.2)	50 (62.5)	0.15
Atrial fibrillation	167 (20.1)	23 (28.8)	0.07
Diabetes mellitus	198 (23.9)	20 (25)	0.82
Obesity	132 (15.9)	12 (15)	0.82
Smoking	114 (13.8)	6 (7.5)	0.11
Drinking alcohol	50 (6)	2 (2.5)	0.19
Hepatic disease	23 (2.8)	3 (3.8)	0.61
Respiratory disease	93 (11.2)	15(18.8)	**0.04**
Chronic renal failure	50 (6)	11 (13.8)	**0.008**
Immunosuppression	23 (2.8)	4 (5.0)	0.26
Previous cardiac surgery	79 (9.5)	11 (13.8)	0.61
Emergent surgery	70 (8.4)	23 (28.8)	**0.0001**
Perioperative factors			
Left ventricle ejection fraction	56.8 ± 11.5	49.6 ± 13.6	**0.0001**
Valve	455 (54.9)	47 (58.8)	0.5
CABG	399 (48.1)	39 (48.8)	0.91
Valve + CABG	109 (13.1)	20 (25.0)	**0.004**
Total CPB time. min	105.3 ± 44.29	156.1 ± 77.7	**0.0001**
Aortic cross-clamp time, min	77.3 ± 37.04	106.9 ± 56.7	**0.0001**
Postoperative value			
Duration of mechanical ventilation, days	25.23 ± 62.59	19.75 ± 31.43	0.81
Reintubation	25 (3)	16 (20)	**0.0001**
Politrasfusion	254 (30.6)	25 (31.3)	0.640
Acute renal failure	67 (8.1)	36 (45.0)	**0.0001**
Reintervención	21 (2.5)	13 (16.3)	**0.0001**
Intra-aortic balloon pump	32 (3.9)	27 (33.8)	**0.0001**
Pneumonia	35 (4.2)	15 (18.8)	**0.0001**
Surgical site infections	25 (3)	1 (1.3)	0.36
Preoperative hospitalization, days	18.2 ± 15.5	10.6 ± 11.3	**0.0001**
Mean ICU stay, days	4.7 ± 12.1	14.3 ± 20.9	**0.0001**
Duration of hospitalization after surgery, days	22.8 ± 20.9	24.9 ± 23.7	0.43
Risk score			
Additive EuroSCORE, points	4.5 ± 2.8	7.4 ± 4.5	**0.0001**
Logistic EuroSCORE, points	5.4 ± 6.2	16.7 ± 18.5	**0.0001**
ACEF score, points	1.2 ± 0.4	1.7 ± 0.6	**0.0001**
SOFA score, points	5.5 ± 1.7	7.7 ± 1.7	**0.0001**
SAP II score, points	28.9 ± 8.7	43.0 ± 11.6	**0.0001**
APACHE II score, points	13.3 ± 3.8	18.3 ± 5.6	**0.0001**

Values are expressed as numbers (*n*), percentages (%), and means ± SD

*APACHE II* Acute Physiology and Chronic Health Evaluation II, *CABG* coronary artery bypass graft, *CPB* cardiopulmonary bypass

**Table 2:** Parameters at the admission to the intensive care unit in survivors and non-survivors

Variable	Survivors (*n* = 829)	Non-survivors (*n* = 80)	*p* value
pH	7.37 ± 0.06	7.31 ± 0.9	**0.0001**
HCO_3_^−^ mEq/l	24.4 ± 3.15	21.04 ± 3.9	**0.0001**
PCO_2_, mmHg	41.0 ± 6.3	41.3 ± 6.2	0.69
PaO_2_/FiO_2,_ range	245.6 ± 72.8	226.2 ± 77.1	**0.024**
Core temperature, °C	36.6 ± 0.8	36.7 ± 1.5	0.22
Leukocyte count, cells/mm^3^	10535.1 ± 6396.8	13454.8 ± 5727.4	**0.0001**
C-reactive protein, mg/L	14.3 ± 35.1	29.5 ± 53.1	**0.014**
Procalcitonin, ng/mL	0.8 ± 4.9	3.1 ± 5.	**0.0001**
Lactates, mmol/L	29.4 ± 14.9	63.6 ± 43.7	**0.0001**
ScvO_2_, %	73.4 ± 8.6	65.6 ± 12.3	**0.0001**
Heart rate, beats/min	92.3 ± 18.5	109.7 ± 17.2	**0.0001**
Mean arterial pressure, mmHg	72.5 ± 13.7	53.6 ± 10.5	**0.0001**
Glucose, mg/dl	181 ± 57.2	193.7 ± 70.5	0.13
Creatinine, mg/dl	1 ± 0.5	1.5 ± 1.3	**0.001**
Hematocrit, %	29.1 ± 4.4	28 ± 4.5	**0.04**
Na, mmol/L	140.1 ± 7.6	142 ± 4.8	**0.028**
K, mmol/L	3.9 ± 0.6	4 ± 0.7	0.47
TnT, μg/l	0.8 ± 1.4	3.8 ± 6.1	**0.0001**
CK-MB, ng/mL	336.4 ± 283.2	161.6 ± 186.4	**0.0001**
INR, range	1.5 ± 0.2	2 ± 0.8	**0.0001**
aPTTr, range	1.2 ± 0.3	1.8 ± 1.2	**0.0001**
Platelet count, cells/mm^3^	119490.8 ± 47226.4	119898.7 ± 65476.3	0.95

Values are expressed as means ± SD

*aPTTr* activated partial thromboplastin time ratio, *CK-MB* creatine kinase-MB, *INR* international normalized ratio, *K* potassium, *Na* sodium, *PaO2/FiO2 ratio* ratio of partial pressure of arterial oxygen to fraction of inspired oxygen, *PCO*_*2*_ partial pressure of carbon dioxide, *ScvO2* central venous oxygen saturation, *TnT* troponin T

**Table 3:** Logistic regression model: risk factors for 90-day In-Hospital Mortality

	B	SE	*p* value	OR	95 % CI
Constant	−11.561	1.155	0.0001	0.0001	
1000/MAP (mmHg)	0.308	0.046	0.0001	1.360	1.242–1.489
1000/Bicarbonate (mEq/L)	0.034	0.013	0.011	1.035	1.008–1.062
Lactate (mg/dL)	0.022	0.007	0.001	1.023	1.009–1.036
INR	1.025	0.397	0.0101	2.788	1.282–6.064

Hosmer-Lemeshow _*X*_^2^ = 8.62; *P* = .37. Multicollinearity diagnostics: 1000/MAP, 0.78 tolerance; 1000/Bicarbonate, 0.87 tolerance; Lactate, 0.64 tolerance and INR, 0.65 tolerance

*B* regression coefficient, *CI* confidence interval, *INR* international normalized ratio, *MAP* Mean arterial pressure, *OR* Odds Ratio

**Table 4:** A new system to predict operative mortality in patients undergoing cardiac surgery: POCAS score

Lactate (mmol/L)	Score	INR	Score
<16.00	3	<1.31	12
16.00 to 24.90	4	1.31 to 1.52	14
25.00 to 34.90	6	1.53 to 1.94	16
35.00 to 52.90	9	1.95 to 2.65	20
53.00 to 79.00	14	>2.65	30
>79.00	21		
MAP (mm Hg)	Score	Bicarbonate (mEq/L)	Score
>75	35	>27.4	14
69 to 75	41	25.7 to 27.4	15
61 to 68	46	24.1 to 25.6	16
60	50	22.8 to 24.0	17
45 to 59	58	20.4 to 22.7	18
<45	75	14.6 to 20.3	22
		<14.6	35

**Table 5:** Development series: predictive function of POCAS score scale for 90-day In- Hospital Mortality (Logistic regression)

	B	SE	*p* value	OR	95 % CI	
POCAS	0.113	0.011	0.0001	1.119	1.096	1.143
Constant	−12.671	1.051	0.0001	0.000		

*B* regression coefficient, *CI* confidence interval, *OR* Odds Ratio

**Table 6:** Operative risk categories with corresponding cumulative risk score for 90-day In-Hospital Mortality after cardiac surgery for the risk groups calculated from the roc curve, cumulative risk score^*^

Risk score	Observed no.	Predicted no.	% probability (95 % CI)	90-Day In-Hospital Mortality (%)	Operative risk categories	% probability (95 % CI)
<75	1	1	0.92 (0.89–0.95)	0.4	Low	2.33 (2.19–2.46)
75–81	3	3	2.10 (2.03–2.16)	1.5		
82–90	11	11	4.82 (4.62–5.02)	6.1		
91–100	12	12	12.99 (12.34–13.65)	11.1	Medium	16.32 (15.25–17.38)
101–105	11	10	25.53 (24.40–26.66)	28.2		
106–128	29	28	56.22 (51.86–60.58)	55.8	High	56.22 (51.86–60.58)
>128	13	13	94.98 (92.75–97.21)	100.0	Very high	94.98 (92.75–97.21)

*CI* confidence interval, *n* number

**Table 7:** Validity of Additive EuroSCORE, Logistic EuroSCORE, ACEF, SOFA, SAP II, APACHE II, and POCAS to predict the 90-day In-Hospital Mortality rate after cardiac surgery with CEC

Criterion for assigning a mortality	AUC [95 % IC]	Ind.Youden Máx	Cutoff value	Sensitivity	Specifity	NPV	PPV	*Χ* ^2^ test AUCs (other scale*POCAS)
POCAS	0.899 [0.864–0.934]*	0.63	85.5	0.91	0.72	98.7	24.9	--
APACHE II	0.768 [0.709–0.828]*	0.42	17.5	0.54	0.88	95.2	30.7	20.42*
SOFA	0.825 [0.782–0.867]*	0.52	5.5	0.92	0.59	98.7	18.8	10.16**
SAPS II	0.847 [0.800–0.893]*	0.56	32.5	0.85	0.71	98.2	20.1	24.10*
ACEF	0.699 [0.639–0.758]*	0.31	1.49	0.50	0.81	93.5	22.3	652.30*
Logistic EuroSCORE	0.754 [0.693–0.814]*	0.40	4.11	0.81	0.58	96.7	17.3	398.28*
Additive EuroSCORE	0.714 [0.647–0.780]*	0.38	5.50	0.71	0.67	95.4	19.2	30.39*

(*) p < 0.0001; (**) p < 0.05

*AUC* area under the curve, *NPV* negative predictive value, *PPV* positive predictive value, *SOFA* Secuencial Organ Failure Assesment, *SAP II* Simplified Acute Physiology Score II, *APACHE II* Acute Physiology and Chronic Health Evaluation II
